# Draft genome sequences of evolutionary distant *Yersinia* species representatives

**DOI:** 10.1128/mra.00996-24

**Published:** 2024-12-10

**Authors:** Amanda Brady, Taylor M. Garrison, Robert K. Ernst, David A. Rasko, Matthew B. Lawrenz

**Affiliations:** 1 Department of Microbiology and Immunology, University of Louisville School of Medicine, Louisville, Kentucky, USA; 2 Department of Microbial Pathogenesis, University of Maryland - Baltimore, School of Dentistry, Baltimore, Maryland, USA; 3 Center for Pathogen Research, University of Maryland - Baltimore, Baltimore, Maryland, USA; 4 Institute for Genome Sciences, University of Maryland – Baltimore, Baltimore, Maryland, USA; 5 Department of Microbiology and Immunology, University of Maryland – Baltimore, Baltimore, Maryland, USA; 6 Center for Predictive Medicine for Biodefense and Emerging Infectious Diseases, University of Louisville, Louisville, Kentucky, USA; The University of Arizona, Tucson, Arizona, USA

**Keywords:** *Yersinia pestis*, Pestoides, genome sequencing

## Abstract

*Yersinia pestis* is the etiological agent of human plague. However, certain evolutionarily divergent subspecies have different host specificities and virulence capacity compared to the more commonly studied strains with pandemic potential. This resource examines 10 diverse isolates representing some of the most understudied subspecies commonly referred to as *Yersinia pestis* Pestoides.

## ANNOUNCEMENT

*Yersinia pestis* is a zoonotic pathogen that causes a lethal infection of humans known as plague. *Y. pestis* evolved from the gastrointestinal pathogen *Y. pseudotuberculosis* (1). During its evolution, several sylvatic plague strains emerged with restricted host range and virulence potential and are considered ancestral to strains with pandemic potential (2, 3). Of these sylvatic plague strains, a group commonly referred to as *Y. pestis* Pestoides provides a unique resource to investigate the genetic diversity within *Y. pestis*. Defining the genetic differences between the *Y. pestis* Pestoides strains and the strains associated with human plague will help understand the evolution of *Y. pestis* and the molecular mechanisms that contribute to the acute virulence of this pathogen.

*Y. pestis* Pestoides strains were isolated from locations within the former Soviet Union prior to 1984 (2, 4). *Y. pestis* Angola was originally isolated from Angola (2). Stabs were obtained from frozen stocks maintained at the Centers for Disease Control and Prevention. From the stab, bacteria were grown overnight in brain–heart infusion (BHI) at 26°C to make glycerol stocks stored at −80°C. All working stocks are less than three passages from frozen stocks.

All work with viable *Y. pestis* strains was performed in select agent-approved BSL-3 laboratories. Bacteria were cultured from frozen stocks on BHI plates for 48 hours at 26°C. From agar plates, multiple colonies were collected to inoculate 5 mL of BHI broth, which were incubated overnight at 26°C on a roller drum for aeration. One milliliter of overnight culture was harvested, and genomic DNA was prepared using the Promega Wizard Genomic DNA Purification Kit. After verification of pathogen inactivation, DNA was provided to SeqCenter (https://www.seqcenter.com/) for production sequencing on the Illumina platform. Sample libraries were prepared using the Illumina DNA Prep kit and IDT 10 bp UDI indices, and sequenced on an Illumina NextSeq 2000, producing 2 × 151 bp reads. Demultiplexing, quality control, and adapter trimming were performed with bcl-convert (v3.9.3) (5) and Trimmomatic v0.39 (6). The number of reads generated for each strain on average was 3,036,663 ± 1,084,806 (Table 1). The reads for each strain were then assembled with SPAdes v3.15.3 (7). The relevant statistics for each genome assembly are listed in Table 1. Default parameters we used with all software, unless otherwise specified. The average genome contained 159.5 ± 18.7 contigs with a complete genome size of 4,571,449 ± 55,878 bp and an average GC% of 47.36±0.43% (Table 1). When the genomes were examined with CheckM v1.2.3 (8), the average completeness was 99.58±0.32% with 0.04±0.03% contamination, indicating that these genomes are of acceptable quality. Genomes were annotated using the NCBI prokaryotic genome annotation pipeline (PGAP v6.8) (9).

**TABLE 1:** *Yersinia* Isolate and Genome Metrics

Species	Strain/Biovar	Isolation location (date)	Grouping^*a*^	Assembly names	Number of read pairs	Number of base pairs	Genome coverage	Contigs	Genome length (bp)	GC%	N50	SRA accession	Assembly accession
*Y. pestis*	*Y. pestis*: KIM5	--	--	Ypestis_KIM5_LOU	2,248,376	337,256,400	73.4	153	4,595,212	46.54	49656	SRR30551973	JBHFDC000000000
*Y. pestis*	pestis: CO-92	Colorado (1992)	--	Ypestis_CO92_LOU	2,412,894	361,934,100	78.2	178	4,627,323	47.51	42191	SRR30551972	JBHFDB000000000
*Y. pestis*	Angola	Angola (pre-1984)	0.PE3	Ypestis_Angola_LOU	2,475,982	371,397,300	83.8	203	4,431,249	47.51	37824	SRR30551971	JBHFDA000000000
*Y. pestis*	Pestoides A	Former Soviet Union (pre-1984)	0.PE1	Ypestis_PestoidesA_LOU	2,354,932	353,239,800	76.7	142	4,603,994	46.54	52729	SRR30551970	JBHFCZ000000000
*Y. pestis*	Pestoides B	Former Soviet Union (pre-1984)	0.PE1	Ypestis_PestoidesB_LOU	2,067,310	310,096,500	67.4	143	4,603,482	47.54	55022	SRR30551969	JBHFCY000000000
*Y. pestis*	Pestoides C	Former Soviet Union (pre-1984)	0.PE1	Ypestis_PestoidesC_LOU	2,783,276	417,491,400	90.8	149	4,597,839	47.55	48595	SRR30551968	JBHFCX000000000
*Y. pestis*	Pestoides D	Former Soviet Union (pre-1984)	0.PE1	Ypestis_PestoidesD_LOU	4,836,188	725,428,200	157.8	162	4,597,021	47.53	46967	SRR30551967	JBHFCW00000000
*Y. pestis*	Pestoides E	Former Soviet Union (pre-1984)	0.PE2.b	Ypestis_PestoidesE_LOU	3,284,588	492,688,200	107.9	152	4,566,422	47.62	48543	SRR30551966	JBHFCV000000000
*Y. pestis*	Pestoides F	Former Soviet Union (pre-1984)	0.PE2.a	Ypestis_PestoidesF_LOU	2,763,258	414488,700	91.2	150	4,546,209	47.62	48958	SRR30551965	JBHFCU000000000
*Y. pestis*	Pestoides G	Former Soviet Union (pre-1984)	0.PE2.b	Ypestis_PestoidesG_LOU	5,139,822	770,973,300	169.6	163	4,545,741	47.59	48857	SRR30551964	JBHFCT000000000

^*a*^ groupings are derived from Achtman et al. (2).

## Data Availability

All data have been released, and SRA and assembly accession numbers are listed in Table 1.
